# A Real-Time 3D Measurement System for the Blast Furnace Burden Surface Using High-Temperature Industrial Endoscope

**DOI:** 10.3390/s20030869

**Published:** 2020-02-06

**Authors:** Tianxiang Xu, Zhipeng Chen, Zhaohui Jiang, Jiancai Huang, Weihua Gui

**Affiliations:** School of Automation, Central South University, Changsha 410083, China; xutx163@163.com (T.X.); jzh0903@csu.edu.cn (Z.J.); huangjiancai@csu.edu.cn (J.H.); gwh@csu.edu.cn (W.G.)

**Keywords:** blast furnaces, industrial endoscope, image enhancement, shape measurement, three-dimensional reconstruction

## Abstract

Capturing the three-dimensional (3D) shape of the burden surface of a blast furnace (BF) in real-time with high accuracy is crucial for improving gas flow distribution, optimizing coke operation, and stabilizing BF operation. However, it is difficult to perform 3D shape measurement of the burden surface in real-time during the ironmaking process because of the high-temperature, high-dust, and lightless enclosed environment inside the BF. To solve this problem, a real-time 3D measurement system is developed in this study by combining an industrial endoscope with a virtual multi-head camera array 3D reconstruction method. First, images of the original burden surface are captured using a purpose-built industrial endoscope. Second, a novel micro-pixel luminance polarization method is proposed and applied to compensate for the heavy noise in the backlit images due to high dust levels and poor light in the enclosed environment. Third, to extract depth information, a multifeature-based depth key frame classifier is designed to filter out images with high levels of clarity and displacement. Finally, a 3D shape burden surface reconstruction method based on a virtual multi-head camera array is proposed for capturing the real-time 3D shape of the burden surface in an operational BF. The results of an industrial experiment illustrate that the proposed method can measure the 3D shape of the entire burden surface and provide reliable burden surface shape information for BF control.

## 1. Introduction

The blast furnace (BF)-based ironmaking process accounts for more than 70% of carbon emissions, and BFs are primarily responsible for the greenhouse gas emissions caused by steel production [1]. Consequently, BF condition and detection of material layer distribution information are key factors for saving energy and reducing emissions. A good burden surface shape can improve gas flow distribution, reduce coke rations, and increase ironmaking efficiency [2,3,4]. However, a BF is a large-scale enclosed reactor, and its operating conditions are characterized by high temperatures, high pressures, and high dust levels. The BF-based ironmaking process involves batching iron ore, coke, and auxiliary materials in a specific proportion, and then blowing hot air into the reactor to facilitate burning of the coke underneath. The harsh environment inside BFs significantly impedes 3D measurement of the burden surface shape. First, it is difficult to capture clear and reliable images of the burden surface because of the high levels of temperature, pressure, and dust in the enclosed environment. Second, image brightness is uneven; the captured images are entirely dark and full of noise, which makes it challenging to process them to obtain clear images. Third, it is challenging to directly apply the traditional 3D measurement method in the complex furnace environment. Thus, a novel method is essential for realizing 3D reconstruction of the BF burden surface in harsh industrial environments.

Several pointwise methods have been employed to gauge the BF burden surface [5,6,7,8,9]. Direct detection methods for shape measurement of the BF burden surface mainly employ mechanical probes, radar probes, laser probes, and infrared imagers. Mechanical probes can perform detection only at discrete times and fixed points. Radar probes capture only the cross-sectional area and average material surface information. Although laser probes have high accuracy, their penetration through dust is poor. Thus, they can operate at their full capacity only during low-dust periods during BF operation and provide poor coke guidance during normal production. Infrared imagers [10,11] are installed atop the BF and are considerably affected by dust. Consequently, they can capture videos of only the central airflow and chute, which contain minimal material information. Other methods have been proposed to obtain the 3D shape of the burden surface. For instance, Dominik Zankl [12,13] proposed a scanning 3D measurement radar with good performance in low-dust environments. However, this radar exhibited poor accuracy in highly dusty environments, which are encountered in typical production periods. 

Several methods have been employed to extract 3D information from a single image sequence. Wu [14,15] proposed a method based on shading reconstruction to obtain the 3D shape information of an object based on shadow cues (gray information) from a single gray image, but this reconstruction process relies solely on an ideal model because the required lighting conditions are very demanding. Given the highly disturbed illumination conditions in BFs, which can be ascribed to the conditions of the light source, it is difficult to obtain fixed lighting strength and direction. Thus, Ramachandran’s method is difficult to use in BFs. F. Tombari [16,17] analyzed a deformed texture unit by projecting an object with a repeated texture unit onto an image and inversely obtained surface normal and depth information about the object for 3D reconstruction. However, the texture distribution of the BF burden surface contains coke ore, fine ore, sinter, and pellets. Thus, the particle size distribution on the surface is not uniform, and the texture distribution is not composed of a repeating texture unit. P. Favaro [18,19] proposed a scheme to restore the depth information of an object by analyzing the relationship between camera focal length and image sharpness with the aim of constructing a 3D model of the object. However, the focusing method requires the structure to be complicated, and the scheme is difficult to use in the complex BF environment.

**Figure 1:**
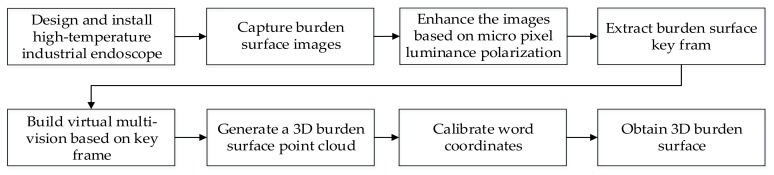
Block diagram of 3D full burden surface measurement system.

To overcome the substantial interference created by the highly dynamic illumination conditions on the burden surface image due to the harsh environment and high dust levels in BFs, a real-time online full BF surface measurement system is developed in this study. The proposed method introduces several techniques to accurately obtain the 3D reconstruction of the burden surface. The overall measurement method shall be read in combination with the process flow shown in Figure 1. This method combines an industrial endoscope with the proposed virtual multi-head camera array 3D reconstruction method. First, to solve the image-acquisition problem, an industrial endoscope is designed and installed to capture images of the actual burden surface. Then, to enhance the burden surface images, polarization of the micro-pixel algorithm is proposed. Finally, to overcome the challenges associated with the extraction of depth information, a method for constructing a virtual multi-head camera array based on depth key frames is proposed, and the realizable shape of the burden surface is clarified substantially.

The remainder of this paper is organized as follows. Section 2 introduces the structure and installation procedure of the industrial endoscope and the proposed clear algorithm. Section 3 describes the proposed method for 3D reconstruction of the burden surface. Section 4 discusses the experimental results. Section 5 presents the conclusions.

## 2. Image Acquisition of BF Burden Surface Using Industrial Endoscope

To capture the shape of the BF burden surface, a novel device capable of capturing images of the burden surface is the first requirement. Then, the sharpness of the originally captured burden surface images is analyzed, and the CLEAR algorithm, which is based on micro-pixel luminance polarization, is proposed. The details are as follows.

### 2.1. Introduction of Burden Surface Imaging System Based on Industrial Endoscope

Considering the high-temperature, high-pressure, high-dust, and low-light environment inside a BF, a parallel low-light-loss backlit industrial endoscope was developed to capture a video of the burden surface. A parallel low-light-loss backlit path and a low-temperature cold light source at the back end were used to overcome the challenges created by poor lighting in the closed BF environment. To ensure functioning of the equipment at high temperatures, the high-temperature-resistant lens was placed separately from the imaging chip, which is not resistant to high temperatures, by following the principle of optical fiber guiding. Furthermore, the air- and water-cooled double-circuit structure was employed to realize safety, ease of installation, and online maintenance of the equipment, in addition to equipment cooling. 

**Figure 2:**
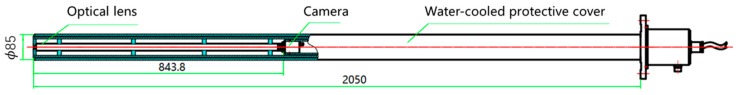
Schematic diagram and dimensions of the cover.

**Figure 3:**
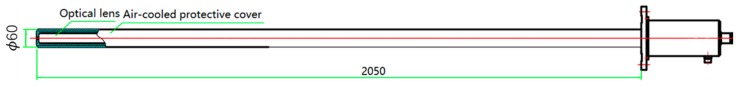
Schematic diagram and dimensions of the internal structure.

**Figure 4:**
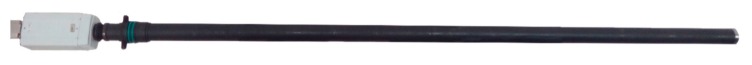
Photograph of the imaging component.

**Figure 5:**
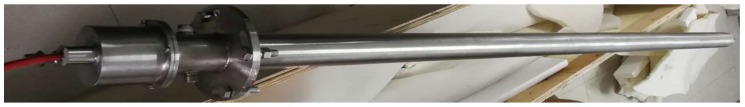
Photograph of the high-temperature industrial endoscope.

The parallel low-light-loss backlit industrial endoscope used in this study is mainly composed of four functional components: an imaging component, a backlight component, protective cooling component, and power component. Schematic diagrams and dimensions of the external and internal parts of the device are shown in Figure 2 and Figure 3, respectively. The imaging component is mainly composed of an imaging tube and an imaging lens. The backlit assembly is mainly composed of a backlit tube. The protection cooling assembly protect the sleeve by using the double cooling assembly, as shown in Figure 4. The power supply components mainly include the power supply and imaging drive circuits, as well as connecting lines for device power supply and video signal transmission. A photograph of the industrial endoscope used in this study is shown in Figure 5. The endoscope works as follows. First, the cooling assembly ensures that the protection device can function in the high-temperature, high-pressure environment inside the BF. Then, the backlit assembly within the device uses light from an external source to provide sufficient illumination in the furnace for the imaging assembly at the front end. Once again, the imaging tube of the imaging assembly exports the captured BF surface images to the low-temperature region at the back end of the device digitally images the imaging chip of the imaging tube and its imaging driving circuit. Finally, the video signal line interface of the power component is realized. Digital video information of the BF burden surface is transmitted to complete the entire workflow.

**Figure 6:**
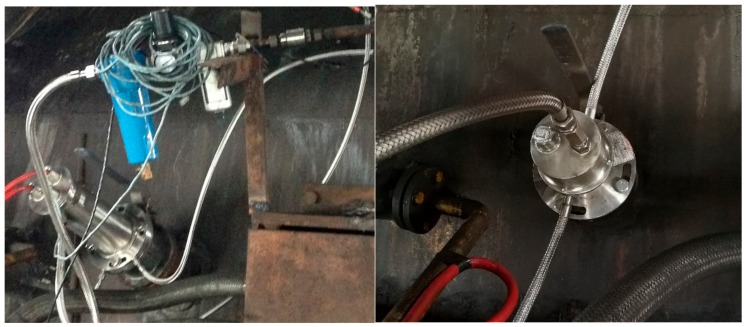
Installed industrial endoscope.

For the proposed burden surface imaging system based on the industrial endoscope, reasonable installation of the industrial endoscope is essential for achieving long-term operation and high imaging quality. First, the equipment needs to be installed in a low-dust area to reduce the influence of dust on image quality. This study follows the BF top dust movement distribution outlined in [20] to determine the location of the low-dust area. Second, the front end of the equipment should be located 1–1.5 m away from the material surface to ensure that the backlight provides an adequate level of brightness. Third, the device should be installed less than 1 m away from the inner furnace wall while ensuring that it is in a region through which no material flows with a sufficient safety margin, so that the device is not hit by the stream. Figure 6 shows the installed industrial endoscope.

**Figure 7:**
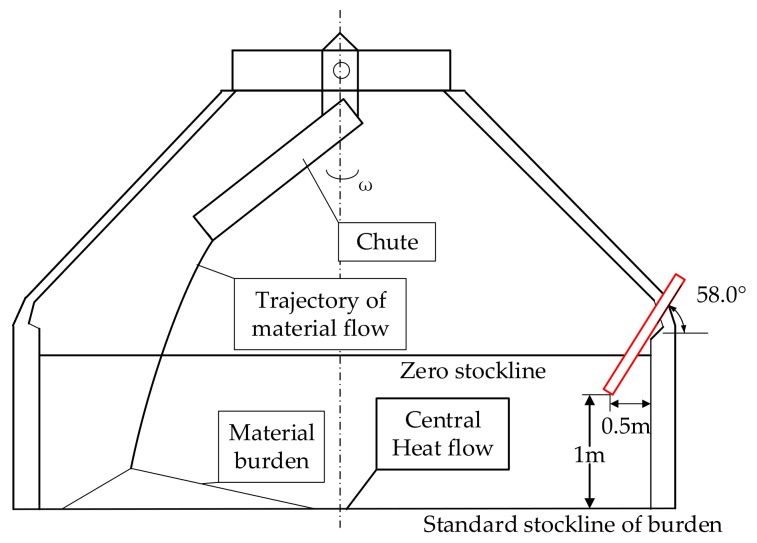
Schematic diagram of installation of high-temperature industrial endoscope.

The device is installed at the position shown in Figure 7, such that it makes an angle of 58° with the horizontal. The distance between the front end of the device and the standard material line is 1 m, and the distance between the front end of the device and the inner wall of the furnace is 0.5 m. Thus, the device lens directly captures the center airflow and the material surface. The part of the equipment outside the furnace is connected to the air- and water-cooled passage and the power–data transmission line. Therefore, videos of the material drop can be captured in real-time.

### 2.2. Construction of Key Frame Classifier

**Figure 8:**
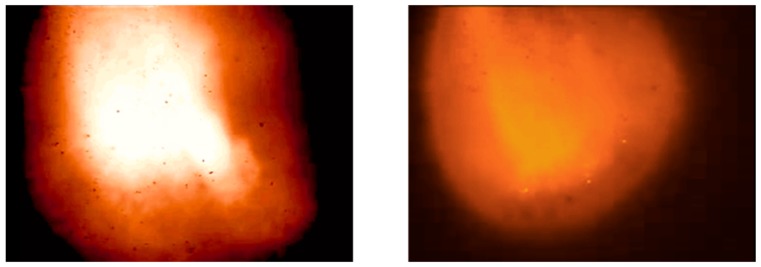
A few low-quality images of the burden surface.

**Figure 9:**
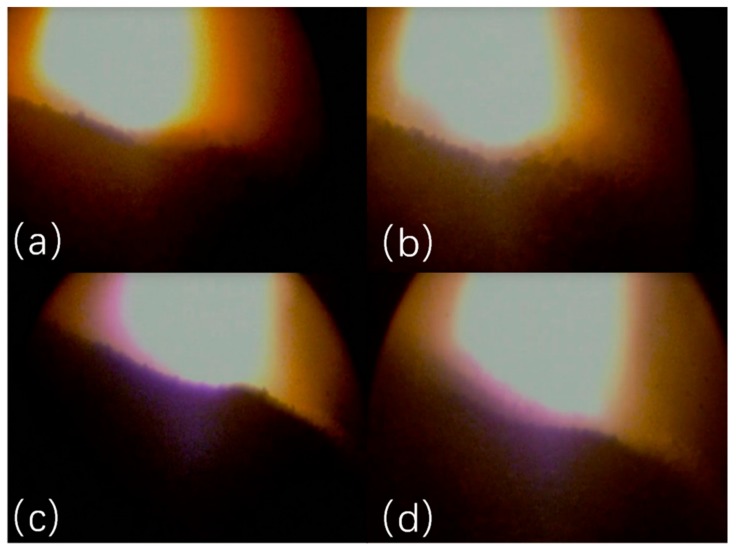
Bad Burden surface images captured using the industrial endoscope. (**a**) Unclear texture image. (**b**) Low edge strength image. (**c**) Low image brightness image. (**d**) Low signal-to-noise ratio image.

In the burden surface images captured using the industrial endoscope, not every image frame is clear and rich in details. A few low-quality images are obtained, as shown in Figure 8. The images in Figure 8 depict only a large flame in the center and marginal amounts of material information. These burden surface images cannot be used for 3D reconstruction. Figure 9 shows a few burden surface images captured using the industrial endoscope, Figure 9a,b is captured when the burden surface is even and average lighted, Figure 9c,d is captured when the burden surface is tilted and not well lighted. However, the sharpness, brightness, and material information of these images are different. In the images in Figure 9a,b, texture details are unclear, and edge strength is low. The images shown in Figure 9c,d is characterized by low image brightness and signal-to-noise ratio, and graininess of the material is unclear. Therefore, these images were filtered out during image processing.

The BF level drop process is slow. Video sequences captured during a falling plane typically contain more than 9000 highly overlapping frames. Therefore, one must judge whether the candidate key frames can actually be used as key frames. During falling of the charge, the central airflow area in the video sequence is overexposed, central airflow is unstable, and the key frame cannot be satisfied simply by a fixed interval. In the videos captured inside the BF, image details are very important, and the central airflow area cannot be changed rapidly because doing so would destabilize feature tracking.

**Figure 10:**
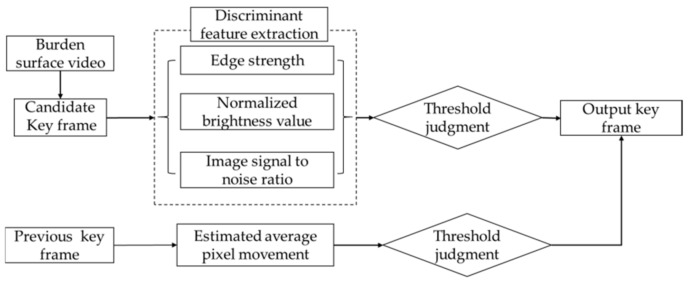
Key frame discriminator for burden surface videos.

Image description is often performed using descriptors of the target features, which represent the characteristics of the target image. An important task in image analysis is obtaining quantized values of the target features from an image. Features for image recognition can pass through edges, outlines, textures, and regions of the image, all of which are intuitive features of image grayscale. Their physical meanings are clear, extraction is relatively easy, and the corresponding extraction algorithm can be designed for specific problems. Thereafter, the designed algorithm can be used as an input training classifier. For the image grayscale features, edge intensity is considered, luminance valued are normalized, and amount of noise is considered the image sharpness feature. The key frame discriminator designed for the burden surface videos captured using the industrial endoscope is shown schematically in Figure 10.

(1) Edge intensity can reflect image clarity. The larger the value, the definition of the image sharpness based on the Laplacian gradient function is as follows,

(1) $$D(f)=\sum_{y}\sum_{x}\left|G(x,y)\right|$$

where $G\left(x,y\right)$ is the convolution of the Laplacian operator at pixel $\left(x,y\right)$.

(2) Luminance value is normalized, and uniform luminance of the image is defined as the sum of the pixels exceeding the high-luminance threshold and low-luminance threshold. Superimpose to obtain an image with uniform luminance distribution.

(2) $$Lum_{avg}=e^{\frac{1}{N}\sum_{X,Y}\ln(\delta +lum(x,y))}$$

(3) Noise is a high-frequency component, and the image edges are high-frequency components with high gradient values. Nonetheless, the image edges have clear structural features. According to these features, image noise can be separated from image edges. In this study, the Tenenbaum gradient function is modified such that the original vertical and horizontal gradients are transformed into four-direction gradients. Tenenbaum gradient function convolves an image with Sobel operators, and then sums the square of the gradient vector components. After the image is convoluted with the Sobel operators to gain four-direction gradients, the minimum of the four directional gradients is defined as the final gradient of a pixel. When the gradient of a pixel is greater than a preset threshold, the pixel is classified as a noise pixel.

(3) $$G(x,y)=f(x,y)*S_{k}(x)\;k=1,2,3,4$$

where G(x,y) is the gradient value,f(x,y) is the pixel value at the coordinates (x,y), and $g_{k}(x,y)$ is the direction operator that is defined as

(4) $$\begin{array}{l} s_{1}\left(x,y\right)=\left[\begin{array}{ccc} -1 & 0 & 1\\ -2 & 0 & 2\\ -1 & 0 & 1 \end{array}\right]{,\;s}_{2}\left(x,y\right)=\left[\begin{array}{ccc} 1 & 2 & 1\\ 0 & 0 & 0\\ -1 & -2 & 1 \end{array}\right]\\ s_{3}\left(x,y\right)=\left[\begin{array}{ccc} 2 & 1 & 0\\ 1 & 0 & -1\\ 0 & -1 & -2 \end{array}\right]{,\;s}_{4}\left(x,y\right)=\left[\begin{array}{ccc} 0 & 1 & 2\\ -1 & 0 & 1\\ -2 & -2 & 0 \end{array}\right] \end{array}$$

(4) Image Displacement Features: To ensure that the acquired key frames can be used for 3D reconstruction, a certain baseline distance should be maintained between the key frames used in the calculation. It is necessary to consider the displacement between a given frame and the previous frame. In this study, a relative frame-based optical flow method was developed to estimate the displacement of the blast furnace surface in images. The proposed optical flow method, which adds the magnitude of the optical flow value of each frame pair and normalizes the sum against the total image displacement sum, was used because it outperformed other optical flow methods in this object application. The normalized pixel displacement input classifier was used as the image pixel displacement feature to ensure that the total pixel displacements of the images were not excessively small, and for making threshold judgments.

Assume that the local light flow and gradient are constant, that is,

(5) $$\forall y\in N(x),d=\frac{\partial X}{\partial t}$$

(6) $$\frac{d}{dt}\nabla E(X,t)=\frac{\partial \nabla E}{\partial X}\frac{\partial X}{\partial t}+\frac{\partial \nabla E}{\partial t}=H(E)\cdot d+{\left(\nabla E\right)}_{t}=0$$

where *E* represents the input image and *X* is a variable that represents the 2D coordinates of the image. Thus, we have

(7) $$X={\left(x,y\right)}^{T}$$

(8) $$E(X)=X^{T}A_{1}X+b_{1}{}^{T}X+c_{1}$$

combine it, we have

(9) $$E(x,y)=r_{1}+r_{2}x+r_{3}y+r_{4}x^{2}+r_{5}y^{2}+r_{6}xy$$

where $c=r_{1},b=\left(\begin{array}{c} r_{1}\\ r_{3} \end{array}\right),A=\left(\begin{array}{cc} r_{4} & r_{6}/2\\ r_{6}/2 & r_{5} \end{array}\right)$.

Suppose that the displacement between the current frame and the previous key frame is d. Then, the current frame image can be expressed as follows.

(10) $$\begin{array}{l} E(\bar{X})=E(X-d)\\ \;\;={\left(X-d\right)}^{T}A_{1}X+b_{1}{}^{T}(X-d)+c_{2}\\ \;\;=X^{T}A_{1}X+b_{1}-2A_{1}d^{T}X+d^{T}Ad-b_{1}{}^{T}d+c_{1}\\ \;\;=X^{T}A_{2}X+b_{2}{}^{T}X+c_{2} \end{array}$$

In the above expression,

(11) $$\left\{\begin{array}{c} A_{2}=A_{1}\\ b_{2}=b_{1}-2A_{1}d\\ c_{2}=d^{T}A_{1}d-b_{1}{}^{T}d+c_{1} \end{array}\right.$$

Thus, the pixel shift displacement can be calculated as

(12) $$d=-\frac{1}{2}A_{1}{}^{-1}(b_{2}-b_{1})$$

Because the image of the falling burden surface cannot simply be considered a binary polynomial, as for the amount of displacement of the burden surface, approximate the true value of *A*(*X*) by averaging.

(13) $$A(X)=\frac{\left(A_{1}X+A_{2}X\right)}{2}$$

(14) $$\Delta b(X)=-\frac{1}{2}(b_{2}(X)-b_{1}(X))$$

Then, an objective function is constructed to optimize the displacement as follows.

(15) $$\sum_{\Delta X\in I}w(\Delta X){\left\|A(X+\Delta X)d(X)-\Delta b(X+\Delta X)\right\|}^{2}$$

Thereafter, through

(16) $$S=\left(\begin{array}{cccccccc} 1 & x & y & 0 & 0 & 0 & x^{2} & xy\\ 0 & 0 & 0 & 1 & x & y & xy & y^{2} \end{array}\right)$$

(17) $$p={\left(a_{1}\;a_{2}\;a_{3}\;a_{4}\;a_{5}\;a_{6}\;a_{7}\;a_{8}\right)}^{T}$$

we have

(18) $$\sum_{i}w_{i}{\left\|A_{i}S_{i}p-\Delta b_{i}\right\|}^{2}$$

where *i* denotes the index of each pixel in the field.

(19) $$p={\left(\sum_{i}w_{i}S_{i}{}^{T}A_{i}{}^{T}A_{i}S_{i}\right)}^{-1}\sum_{i}w_{i}S_{i}{}^{T}A_{i}{}^{T}\Delta b_{i}$$

The displacement vectors along the x and y directions can be obtained. In Equation (17), only Δb is unknown. To find the solution of Δb, an a priori estimate $\bar{d}(X)$, which is an estimate of the true displacement, is introduced in this study.

(20) $$\bar{X}=X+\bar{d}(X)$$

By combining Equations (20) and (14), we have

(21) $$A(X)=\frac{A_{1}X+A_{2}(\bar{X})}{2}$$

(22) $$\Delta b(X)=-\frac{1}{2}(b_{2}(\bar{X})-b_{1}(\bar{X}))+A(X)\bar{d}(X)$$

First, $\bar{d}(X)$ is set to 0, and a new displacement value is calculated using Equation (22). This displacement is more accurate than the previous one, and this new displacement value is substituted as the new value of $\bar{d}(X)$. After multiple iterations, the exact displacement value is obtained.

**Figure 11:**
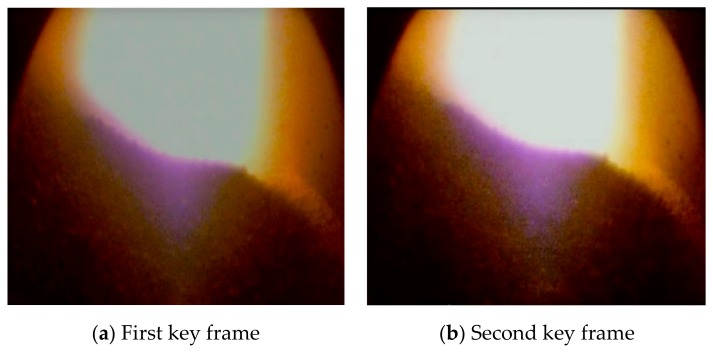
Selected key frames.

The output of the proposed key frame classifier is shown in Figure 11. Compared with the burden surface image in Figure 9, the key frame has stronger edges, higher brightness, and higher signal-to-noise ratio. Moreover, when the first frame key frame is selected, the second frame key frame selected by the discriminator has a large pixel movement. Figure 11 shows the existence of a certain baseline distance between the given frame and the previous frame.

### 2.3. Burden Surface Image Sharpening Algorithm Based on Micropixel Brightness Polarization

#### 2.3.1. Industrial Endoscope Image Clarity

**Figure 12:**
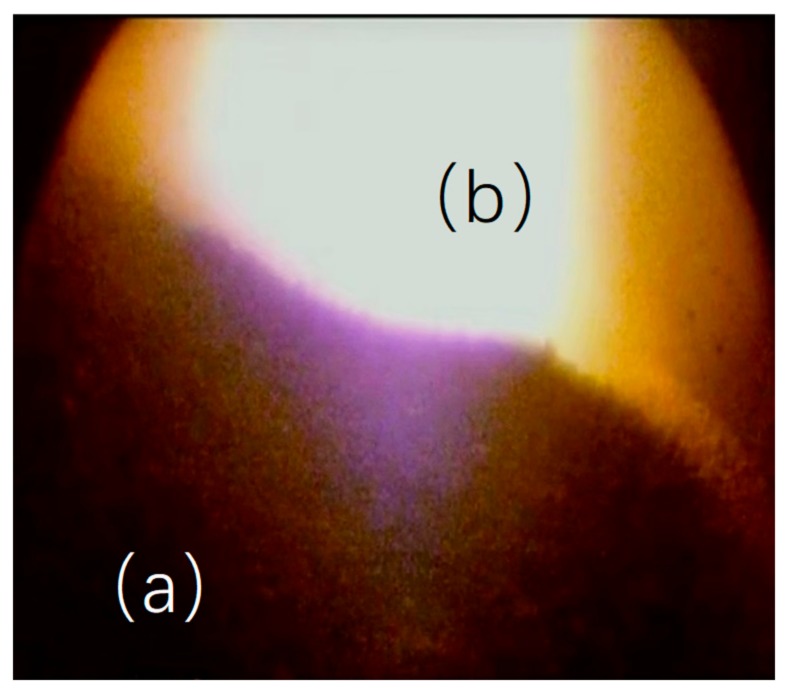
Polarized area in burden surface image. (**a**) Polarized dark region. (**b**) Polarized bright region.

During the material discharge process, the light source of the central gas flow is easily blocked by the material peak. Consequently, the overall video of the burden surface is dark, and the central gas flow is easily overexposed. The brightness of the image is polarized, and the image contains both overexposed and overly dark regions. Owing to the influence of dust in the furnace, the image is very noisy. The material burden contains multiple burden loops, and a non-single burden loop contour distribution is formed. The polarized area of the burden surface image is shown in Figure 12. Figure 12a area represents the polarized dark region that is focused on the material and away from the highlight area. Figure 12b area represents the polarized bright region that is focused on the highlight area.

The characteristics of the BF burden surface video lead to the following difficulties from the viewpoint of achieving the desired image clarity: (a) The noise in the video makes it more difficult to clear up the video, and when the brightness is poor, it is difficult to filter out noise from the image detail features while preserving the texture details. (b) Extremely bright and extremely dark areas are difficult to segment, extremely bright edges are ambiguous, central airflow is directed opposite to the material surface and is affected by airflow disturbances in the BF, and jitter is large. (c) The requirements of the sharpening algorithm in the extremely bright and extremely dark areas are contradictory, that is, the algorithm needs to suppress brightness in the extremely bright areas, whereas it needs to enhance detail and increase brightness in the extremely dark areas. In summary, image subregions are sharpened to ensure that the brightness and sharpness of the material falling process sequence are consistent. This is beneficial for tracking the depth characteristics of the burden ring, which is a prerequisite for 3D reconstruction.

The high-gloss area can be ascribed to the high-temperature airflow at the center of the BF. Because of the high-temperature airflow and splashing of the charge, some areas of the image are extremely bright, and image details in these areas are suppressed. Therefore, suppressing the highlight areas is important for ensuring consistent surface reflection characteristics and sharpness.

**Figure 13:**
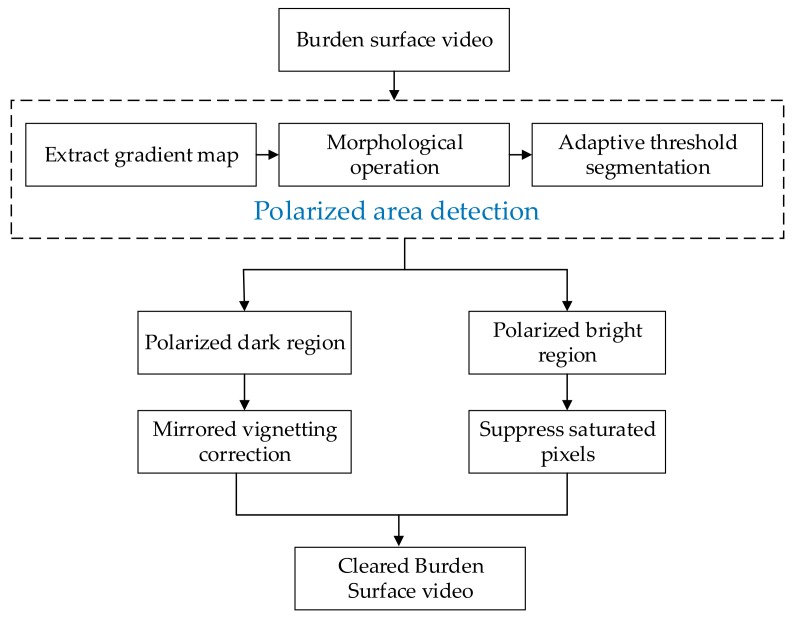
Micro-pixel luminance polarization method.

A block diagram of the micro-pixel luminance polarization method developed in this study is shown in Figure 13. The proposed sharpening algorithm performs highlight detection and suppression and increases the brightness of the surrounding dark regions by using the micro-pixel luminance polarization method. For realizing detection of the luminance polarization region, we propose a method for determining the polarization region by using gray information. This method simultaneously computes image grayscale. An adaptive threshold that can be used to identify the highlight region is determined based on the average grayscale value and the standard deviation of the gray image.

#### 2.3.2. Principle of Micro-Pixel Luminance Polarization

A micro-pixel is a set of pixels that have morphological consistency and continuous brightness. A micro-pixel uses the same quantization step size and motion matrix to form the grayscale polarization region, and it takes square area pixels. When the BF material surface is subjected to luminance polarization division, image details are enhanced for the characteristics of the micro-pixel. Then, the polarization area is tracked to ensure the correctness of block matching. The micro-pixel quantization formula is given in Equation (23)

(23) $$m=\frac{1}{N}\sum_{(i,j)\in R}\left(f(i-1,j\right)+f(i,j)+f(i+1,j))$$

where *m* is average value of the squares in the micro-pixel region.

First, the continuous frame average pixel is calculated. Then, the inter-frame moving pixels are taken as $V_{i}$. The sum of the micro-pixel moving speeds is $V_{0}$, and the predicted value of change in the velocity of the micro-pixel in the polarization region can be obtained as follows.

(24) $$V_{0}=w_{i}V_{i}$$

(25) $$\mathrm{mask}I_{highlight}=\left\{\begin{array}{c} 1,\;\;I<\mu +\sigma \\ \;0,\;\;otherwise \end{array}\right.$$

where $\mu_{1}$ and $\sigma_{1}$ denote the grayscale mean and standard deviation of the grayscale images, respectively.

When the highlight area is detected, the number of saturated pixels can be reduced by replacing the highlight region with the intensity value derived from the combination of adjacent pixels’ values. In this manner, the compensation value of the highlight edge region can be obtained.

(26) P(p)=C(p)∗D(p)

(27) $$\left\{\begin{array}{c} C\left(p\right)=\frac{\sum_{q\in \varphi_{p}\cap \phi }C(q)}{\left|\varphi_{p}\right|}\\ D(p)=\frac{\left|\nabla I\frac{1}{P}n_{p}\right|}{\alpha } \end{array}\right.$$

where C(p) determines whether a point requires compensation in the highlight area $\varphi_{p}$, D(p) is compensation value calculated by the pixel value it is adjacent to. The compensation adjustment value can be computed as follows:

(28) $$SSD(p,q)=\arg\;\mathrm{minSSD}(\varphi_{p},\varphi_{q})$$

(29) $$SSD(p,q)=\sqrt[{3}]{\sum_{i=1}^{m}\sum_{j=1}^{n}\left[{\left(p^{R}{}_{ij}-q^{R}{}_{ij}\right)}^{2}+{\left(p^{G}{}_{ij}-q^{G}{}_{ij}\right)}^{2}+{\left(p^{B}{}_{ij}-q^{B}{}_{ij}\right)}^{2}\right]}$$

where R, G, and B denote the red, green, and blue colors when finding the optimal matching compensation, respectively. The optimal match is found when the average pixel value difference between the highlighted micro-pixel $\varphi_{p}$ and the compensation micro-pixel $\varphi_{q}$ is the minimum. The three color components comprise the compensation block, and the uneven illumination distribution at the image center is processed by applying the radial gradient forced symmetry method. In this study, the vignette correction method is applied to strengthen the dark areas around the image by forcing the symmetry of the radial gradient from the center toward the boundary.

First, background information is extracted by using variance information of the scene. Then, the background gray distribution is approximated using a binary cubic polynomial, and the distribution of the entire image plane correction factor is calculated. Correction of the vignette effect is realized by processing each image frame.

The incident light beams parallel to the lens are collected at the center of the image plane at point A. Assuming that the illuminance at this point is $I_{A}$, light beams that make an angle w with the optical axis are concentrated in image $I_{B}$.

(30) $$I_{B}=KI_{A}\cos4\omega$$

where *K* is the fading coefficient.

By analyzing the histogram of the image, we can determine whether the target and the background have independent gray distribution intervals. By setting a reasonable threshold, we can segment the target and the background. The specific operation method is as follows.

Step 1: Take a frame of size *k* from the image, find the average *k* value of each pixel in the frame $aver\left(I\right)$, and then compute the variance of each pixel var(i,j).

Step 2: Calculate the global threshold.

(31) $$\mathrm{thre}=3\frac{\mathrm{sum}(\mathrm{var}(i,j))}{M\times N}$$

Step 3: Calculate the background pixels.

(32) $$I_{\mathrm{bg}}(i,j)=I(i,j),\begin{array}{l} if\;var(i,j)\leq thre \end{array}$$

Step 4: Refer to the background gray distribution model obtained by approximation.

(33) $$f(x,y)=p_{00}+p_{10}x+p_{01}y+p_{20}x^{2}+p_{11}xy+p_{02}y^{2}+p_{30}x^{3}+p_{21}x^{2}y+p_{12}xy^{2}+p_{03}y^{3}$$

Step 5: Compensate for the vignette effect.

(34) $$F_{CF}(i,j)=f_{max}(i,j)/f(i,j)$$

## 3. Method for Constructing Blast Furnace Surface by Using Virtual Multi-Head Camera Array Based on Key Frames

**Figure 14:**
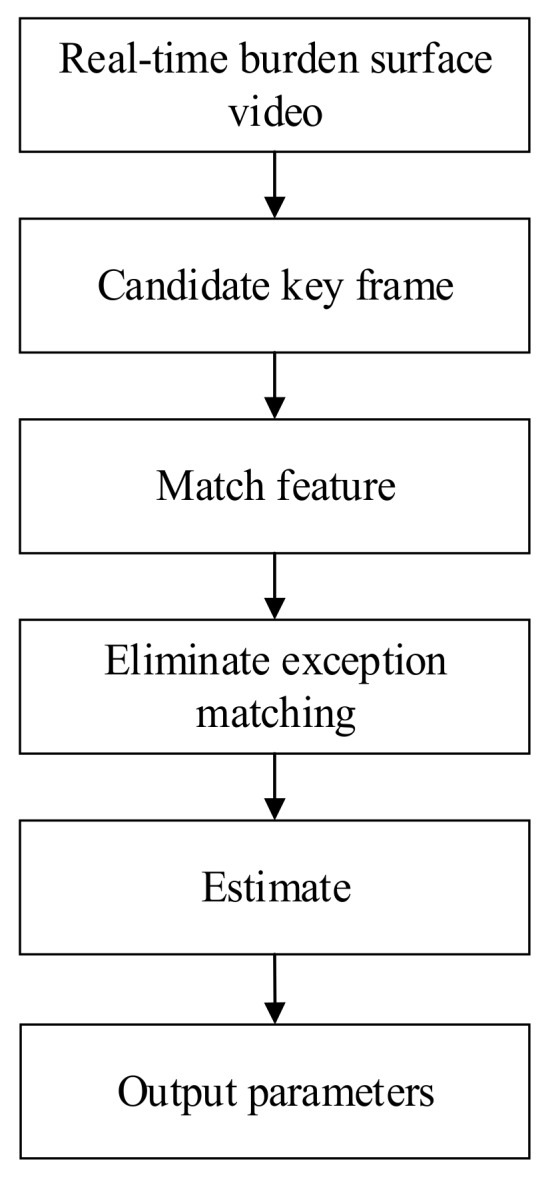
Overall block diagram of the virtual multi-head camera array construction.

In this section, the proposed method for 3D measurement of the BF burden surface from industrial endoscope images is introduced systematically. First, the virtual multi-head camera essential matrix is estimated for constructing camera pairs of the corresponding depth key frames. Then, the BF burden surface shape is reconstructed from the image pairs constructed using the virtual multi-head camera parameters. A block diagram of the virtual multi-head camera array construction method developed in this study is shown in Figure 14. Finally, the BF burden surface shape is scaled to the world coordinate system by using mechanical rod data and radar data.

### 3.1. Acquisition of Essential Matrix of Virtual Multi-Head Camera

Based on the characteristics of the slow surface change process, space-time multiplexing of the monocular endoscope can yield 3D information about the BF burden surface when relative movement occurs between the BF burden surface and the industrial endoscope. The construction of a virtual multi-head camera array is vital from the viewpoint of obtaining this information for 3D detection of the BF. The key frame of the virtual multi-head camera array constructed for the BF material surface captures a high-quality image subsequence of the entire process of falling surface movement in the direction of relative motion between the material surface and the industrial endoscope lens in the burden cycle. After obtaining the key frame, the camera and the surface relative attitude of the key frame can be estimated. The BF estimation method includes three processing steps: detection of feature points, determination of correspondence, and estimation of camera motion parameters. The ORB (Oriented FAST and Rotated BRIEF) feature-point detector is used to detect feature points with sub-pixel accuracy. ORB builds on the well-known Features from Accelerated Segment Test (FAST) keypoint detector. ORB algorithm uses a multiscale image pyramid to gain orientation component and multiscale features. By comparing the hamming distance of the feature-point descriptions between adjacent frames, one can roughly match the list of two consecutive key frames and the feature points in $L_{C}$.

Assume the feature point coordinate set and the image coordinates of the feature points as follows, respectively.

(35) $$\begin{array}{l} L=\{p_{{}^{1}},\cdots ,p_{{}^{i}},\cdots ,p_{{}^{m}}\}\\ p_{i}=(x_{i},y_{i}) \end{array}$$

To estimate the camera motion parameters from the corresponding feature points, the real camera must be represented using a mathematical camera model. The desired relationship between the corresponding feature points in two images follows the surface–camera relative motion model.

(36) $$Z_{C}\left[\begin{array}{c} x\\ y\\ 1 \end{array}\right]=\left[\begin{array}{ccc} f_{x} & 0 & c_{x}\\ 0 & f_{y} & c_{y}\\ 0 & 0 & 1 \end{array}\right]\left[\begin{array}{cc} R & t \end{array}\right]\left[\begin{array}{c} X\\ Y\\ Z\\ 1 \end{array}\right]$$

A is an internal parameter matrix used to represent the conversion relationship between the pixel coordinate system and the camera coordinate system, and the outer parameter matrix [R,t] is used to represent the relationship between the camera coordinate system and the world coordinate system.

There is a correspondence between the same feature point in the previous key frame $P\prime \left(X,Y,Z\right)$ and the next key frame P(X,Y,Z). The relationship between the two key frames is expressed as $P^{\prime }=R\left(P-C^{\prime }\right)$

(37) $$\begin{array}{l} t={\left(t_{X},t_{Y},t_{Z}\right)}^{T}=-RC\prime \\ P=RP+t \end{array}$$

(38) $$x_{i}^{’}=\frac{x_{i}+f\frac{t_{x}}{z_{i}}}{1+\frac{t_{z}}{z_{i}}},y_{i}^{’}=\frac{y_{i}+f\frac{t_{y}}{z_{i}}}{1+\frac{t_{z}}{z_{i}}}$$

By combining Equations (37) and (38), the camera translation relationship can be derived as follows.

(39) $$\begin{array}{l} y_{i}^{\prime }-y_{i}\frac{t_{x}}{t_{z}}-x_{i}^{\prime }-x_{i}\frac{t_{y}}{t_{z}}+\frac{\left(x_{i}^{,}y_{i}-x_{i}y_{i}^{,}\right)}{f}=0\\ where\sqrt{\;\;t_{x}^{2}+t_{y}^{2}+t_{z}^{2}}=k \end{array}$$

A few characteristic point pairs have matching errors because of mismatch. To increase the rate of correct matching, the random sampling method is used to detect outliers, randomly select a set of matching outliers for the calculation and validation matching sets, and repeat iterations until the errors in the validation match set are small.

(40) $$\begin{array}{l} \underset{i}{\sum }\xi_{i}(t_{x},t_{y},t_{z})\to minimize\\ \xi_{i}(t_{x},t_{y},t_{z})=y_{i}^{\prime }-y_{i}\frac{t_{x}}{t_{z}}-x_{i}^{\prime }-x_{i}\frac{t_{y}}{t_{z}}+\frac{\left(x_{i}^{,}y_{i}-x_{i}y_{i}^{,}\right)}{f} \end{array}$$

### 3.2. Construction of Virtual Camera Pairs

3D reconstruction of the contour of the BF is to convert the contour space information (the contour feature of the material on the burden ring) into a spatial data field and then extract the isosurface according to the triangulation method to obtain the 3D model of the material surface. After the camera-surface relative pose matrix is obtained, triangulation is performed by using the same coke ring matching feature set to realize 3D reconstruction. The first step is knowing the camera pose and transformation matrix to world coordinate system. The second part is a mechanical probe scale data scaling, which corrects the 3D absolute coordinates of the high surface charge. BF stock line normal depth generally in the zero stock line below 1.0–1.5 m in a charging cycle, between the stack and the distribution of the receiving level of the stock line, the collision point of impact position and material burden inclination and many other factors, the charge by the collision point rebound.

Due to the BF distribution mechanism and charge distribution, there are similarities in the characteristics of the ten coke rings of the charge. Second, it is necessary to match the feature point pairs of the two images before the parallax is found. However, calculating all the feature points is very time-consuming, so to reduce the matching search range, the feature point set search on the coke ring can be performed. It is possible to quickly reduce the mismatch of nonpolar lines and reduce the set of matching points.

Camera pose is referred to as respect to world reference plane a

(41) $$T_{wc}=\left(\begin{array}{cc} R_{wc} & c_{w}\\ 0^{T} & 1 \end{array}\right)$$

Where $T_{wc}$ is the matrix of camera transfer between camera and world, w is reference frame of the baseline, and *c* is current camera position. Our camera is fixed and intrinsic matrix is calibrated and undistorted before setup. On camera position, $x_{c}=\;(x,\;y,\;z)$ is warped to $\pi (x_{c})\;=\;(x/z;\;y/z)$.

The transformation of key frame respect to the reference frame is

(42) $$s_{1}R_{wc1}x_{1}-s_{2}R_{wc2}x_{2}=c_{w}$$

(43) $$T_{wc}{\left[\begin{array}{cccc} x & y & z & 1 \end{array}\right]}^{T}={\left[\begin{array}{cccc} X & Y & Z & W \end{array}\right]}^{T}$$

According to Equations (41)–(43), the depth of two key frame feature point is

(44) $$T_{wc}=\left[\begin{array}{cccc} 1 & 0 & 0 & -c_{x}\\ 0 & 1 & 0 & -c_{y}\\ 0 & 0 & 0 & f\\ 0 & 0 & -1/T_{x} & \left(c_{x}-c^{\prime }{}_{x}\right)T_{x} \end{array}\right]$$

(45) $$z(x,y)=\frac{T_{wc}-T_{ref}}{X_{R}-X_{T}}$$

where the created 3D model is as close as possible to the contour of the BF burden ring, the theoretical distribution of the BF. Due to the image resolution and the presence of noise, the line of sight is often used as a reconstructed 3D point. Set of two feature points normalized coordinates.

Wherein, respectively, the key frame front principal point coordinate, a focal length and a translation matrix component in the x-axis, for the x-coordinate of the principal point of the right camera. The world coordinates of the space points is calculated by respectively key frame feature.

### 3.3. Scaling Burden Surface to World Coordinate

The 3D BF material surface shape is based on the mechanical probe data to calibrate the 3D shape digital model of the BF burden distribution on surface. The purpose of the BF surface calibration is to accurately determine the 3D shape and height of the BF surface. To correct the high charge surfaces, three mechanical Probes absolute coordinate to correct over the stock line, using actual production data for determining the height of the surface. The 3D digital model of the uncalibrated BF material surface obtained before is used as the basic shape, and the absolute coordinates of the BF coordinate system are obtained after calibration. The radar gauge has low environmental interference and low precision. The mechanical gauge is not affected by smoke and dust, but there is no continuous data. The surface of the material surface is a combination of the two, but the data is preferred when the mechanical probe is effective.

After the calibration, the BF burden surface model is rotated and translated to world coordination, which led to minimum difference between mechanical gauge readings and points of feature correspondence on digital BF burden surface model.

Step 1: Eliminate discrete values: the 3D point cloud to be processed is subjected to noise reduction processing, that is, the outline values are eliminated.

Step 2: Downsampling: 3D point cloud downsampling to be stitched.

Step 3: Select the reference point set: select the newly acquired 3D point cloud as the reference point set P, and the absolute coordinate tracking value of the mechanical probe in the time window is the discrete reference point set M.

Step 4: Calculate the nearest point: for each point in the set P, find the corresponding points closest to the Euclidean distance of the point in the set M, and these corresponding points form a new point set Q.

(46) $${\vec{p}}_{i}=\left(x_{i},y_{i},z_{i}\right),{\vec{q}}_{i}=\left(x_{i},y_{i},z_{i}\right)$$

Step 5: Calculate the rotation translation matrix between point sets P and Q.

(47) $$\begin{array}{l} {\vec{q}}_{i}=R{\vec{p}}_{i}+T+N_{i},i=1,2\ldots N\\ E=\sum_{i=1}^{N}{\left|\left(R{\vec{p}}_{i}+T\right)-{\vec{q}}_{i}\right|}^{2} \end{array}$$

Step 6: Least square method to solve E corresponding to a minimum calculation of coordinate transformation: registration transformation matrix with the collection coordinate is used for transformation to obtain a new set of points.

## 4. Industrial Experiments and Applications

The proposed sharpening algorithm and 3D shape measurement method was tested, validated, and applied at No.2 BF in an ironmaking plant of Liuzhou Steel Co. Ltd., which is the largest iron and steel enterprise in Southwest China. The industrial endoscope was installed in the BF. Through three months of on-site experiments, the sharpening algorithm and 3D shape measurement method did provide clear burden surface images and reliable 3D shape data for BF operators, which were very valuable for reflecting BF operational status and control the BF. The industrial experiments are described in detail in the following.

### 4.1. Performance of the Sharpening Algorithm

**Figure 15:**
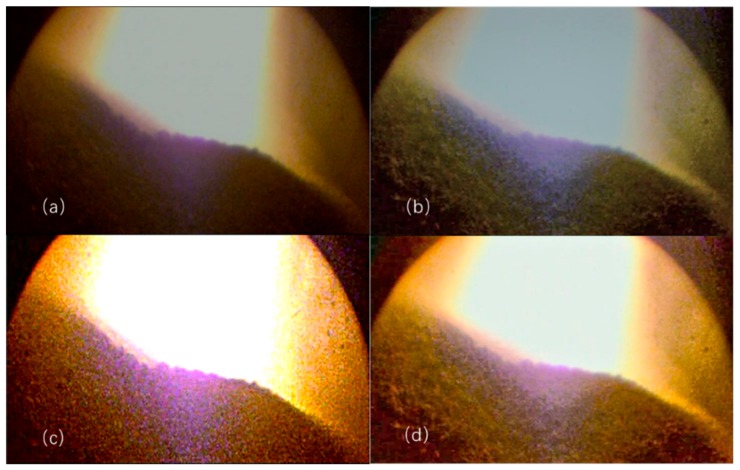
Origin images and clarity results by different methods. (**a**) Original image. (**b**) Result of histogram equalization method. (**c**) Result of retinex method. (**d**)Result of our method.

To show the effectiveness of the sharpening algorithm, compare the proposed clear processing results with the original image before processing under normal furnace conditions. In addition, the method of retinex and histogram equalization are also selected to compare with the proposed method. Figure 15 shows the original image and process result of these three methods. It can be clearly observed that the brightness level of the image after clearing is more uniform, the highlighted area is suppressed, the details of the material surface in the dark are more obvious, and the particles and shape are clearer. Figure 15a represents the original image and overall image is dark, which subject to dust and light double surface blur. Figure 15b is the result of histogram equalization, which has a certain graininess; however, the detail enhancement is not obvious, the contrast is not high, and the outline of the image is more blurred. Figure 15c is the result by retinex, from which it can be seen that the brightness of the image is increased and the contrast is enhanced, but the brightness at the center backlit is unnatural, and the noise is amplified when the texture details are enhanced. The processing results of this method are shown in Figure 15d. It can be clearly observed that the brightness level of the image after clearing is more uniform, the highlighted area is suppressed, the detail of the material surface is more obvious in the dark, the grain and shape are clearer. What is more, from Figure 15d, we can see the shape of the grain of the material surface and the contour of the material surface, the surface of the material caused by the burden ring is undulating.

In addition, to evaluate image quality quantitatively, five indicators are used: image entropy, sum of image power spectrum components, gray mean grads, edge intensity, and point sharpness. The image entropy and sum of image power spectrum components can objectively reflect the volume of image information. The gray mean grads, edge intensity, and point sharpness reflect the image definition. The average value of the quantitative index of 10,320 web surface images under normal furnace conditions is given in Table 1. By comparing Table 1, the power spectral components and image entropy of each image are relatively close, indicating that the image information of the material plane is relatively consistent; however, the calculated values of gray average gradient, edge intensity, and point sharpness are larger than the original image, indicating that the image clarity of the material has been greatly improved compared to the original image; the gray average gradient of the algorithm is 1.99 times, the sharpness of the point is 1.67 times, and the edge intensity is 1.12 times, indicating that the image sharpness is larger improvement. 

### 4.2. Result of 3D Reconstruction

**Figure 16:**
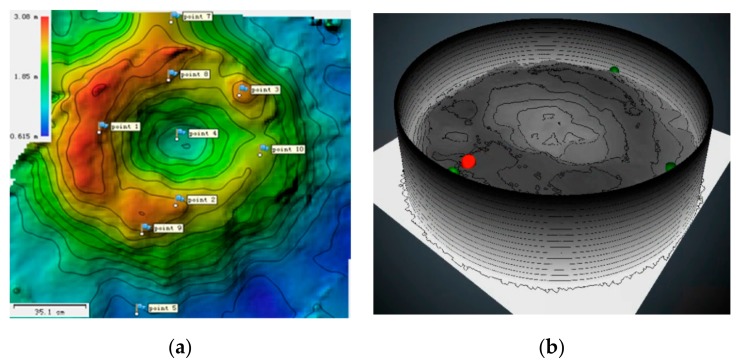
Result of 3D reconstruction. (**a**) Birdview topography 3D map. (**b**) 3D burden surface shape.

Figure 16 shows the result of the 3D reconstruction performed using the proposed method. Figure 16a illustrates the distribution of 10 coke loops, and the corresponding 3D shape is depicted in Figure 16b. In addition, the radar probe (red ball) and mechanical probe (green balls) are marked appropriately. The central region of the burden surface is lower and the edge region is higher, which is consistent with the movement of charge.

**Figure 17:**
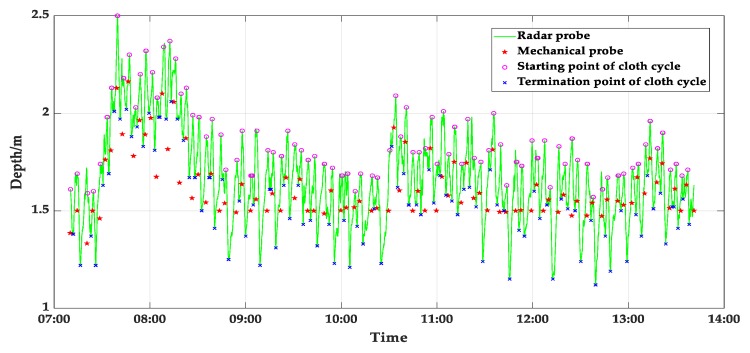
Depth of burden surface as determined using traditional methods.

**Figure 18:**
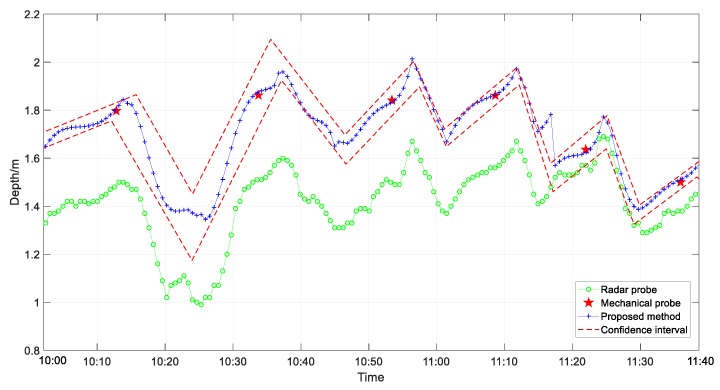
Depth of burden surface as determined using three methods, namely mechanical probe, radar probe, and proposed method.

To verify the accuracy of the 3D reconstruction method, the No.2 BF north surface is used to measure the actual measurement data of the mechanical probe and radar probe in the same area for simulation. The radar frequency was 26 GHz, bandwidth was 1.64 GHz, radar range was 0 to 100 m, and accuracy was ±1%. The radar data were sampled at intervals of 10 s, and the number of data samples was 2338. The mechanical probe data were sampled at intervals of 3–5 min, and number of data samples was 93. Figure 17 shows the measurement results obtained using the radar probe and mechanical probe from 7:00 to 14:00 on one day. The measurement results obtained using the mechanical probe are discontinuous, and the radar probe can measure the height of the material surface in real-time and reflects the trend of changes in the material surface. However, most of the measurement results deviate considerably from those of the mechanical probe. Considering that the mechanical probe is a contact measurement device, and its measurement accuracy is high, as has been confirmed by field workers, the radar probe yielded large measurement errors. Figure 18 shows the depth measurement results obtained using the radar probe, mechanical probe, and the proposed method from 10:00 to 11:40. The 95% confidence interval is marked in the figure. 

**Figure 19:**
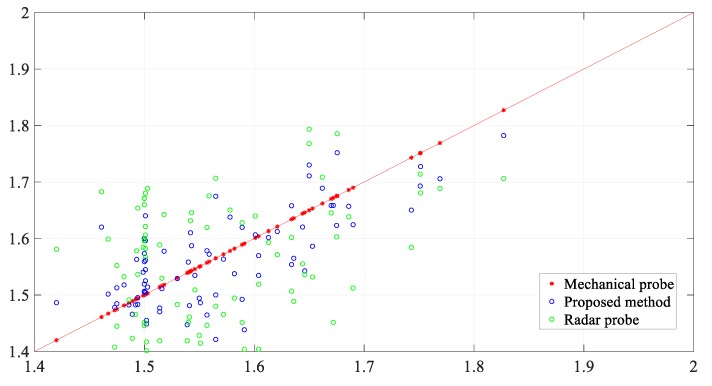
45° line graph and comparison with radar probe.

**Figure 20:**
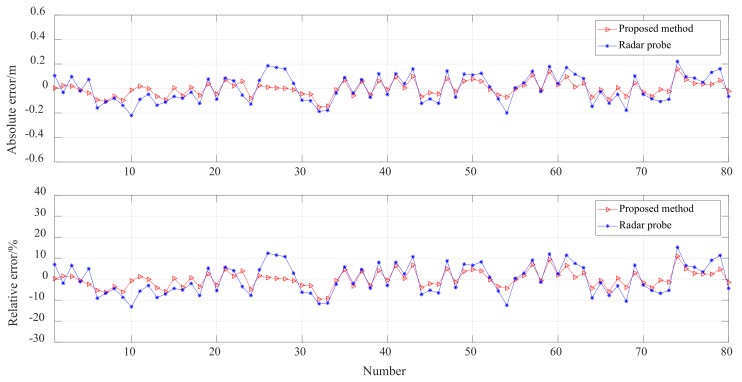
Absolute and relative errors of values obtained using the proposed method and the radar probe.

The result obtained using the proposed method is consistent with the mechanical probe data and consistent with the trend of the radar probe data. Generally, the measurement result obtained using the proposed method is within the confidence interval. Moreover, the measurement data obtained using the mechanical probe, radar probe, and the proposed method were compared. Figure 19 depicts the distribution of the measurement results. Compared with the radar probe data, the values measured using the proposed method change within a smaller true range. The absolute error and relative errors in the values obtained using the proposed method and the radar probe are shown in Figure 20. The absolute error in the values obtained using the proposed method is within the range of ±0.1 m, and the relative error is within the range of ±5%, which means that the accuracy of the proposed method is significantly higher than that of the radar probe.

Furthermore, to prove the universality of the accuracy of the proposed method, three indicators were used: mean relative error (MRE), root mean square error (RMSE), and relative error within 5% (Error-5%). The statistical results obtained using 10,000 data points are summarized in Table 2. The MRE and the RMSE of the proposed method are smaller than those of the radar probe, and the Error-5% of the proposed method is higher than that of the radar probe, which further demonstrates the high accuracy and reliability of the proposed method. Therefore, the proposed method can be used to estimate the 3D shape of the burden surface.

## 5. Conclusions

To continuously measure the 3D shape of the BF burden surface, we proposed a real-time 3D measurement system that employs an industrial endoscope. First, an industrial endoscope was designed and installed at BF No. 2 in an ironmaking plant to capture images of the burden surface during ironmaking. By using a micro-pixel luminance polarization algorithm, details of the captured images were enhanced. Second, the burden surface image key frame was extracted based on the image features and displacement of the burden surface. Thereafter, a virtual multi-vision camera array was constructed to realize 3D reconstruction of the burden surface.

The proposed real-time 3D measurement system was verified in an ironmaking plant, and the measurement results demonstrated its reliability and effectiveness in measuring the 3D shape of the BF burden surface, which can provide critical information for coke operation. Furthermore, the proposed system may spur researchers to work on the 3D reconstruction of objects in high-temperature enclosed environments.

## Figures and Tables

**Table 1:** Quantitative evaluation of image quality with different sharpening methods.

Indicator	Image Entropy	Sum of Image Power Spectrum Components	Gray Mean Grads	Edge Intensity	Point Sharpness
Original image	6.6373	29.3260	1.6142	32.8301	20.0748
Retinex	6.6916	32.7607	2.2253	34.4762	27.9592
Histogram equalization	6.7027	32.3605	2.7347	33.6994	32.7801
Proposed	7.0333	33.3241	3.2255	36.7830	33.4932

**Table 2:** Error comparison of two measurement methods.

Indicator	MRE	RMSE	Error-5%
Radar probe	7.4521%	0.2729	71.51%
Proposed method	4.1453%	0.2036	85.21%

## References

[1] Lin B., Wang X. (2015). Carbon emissions from energy intensive industry in China: Evidence from the iron & steel industry. Renew. Sustain. Energy Rev..

[2] Zhou P., Shi P.-Y., Song Y.-P., Tang K.-L., Fu N., Zhou C.Q. (2016). Evaluation of Burden Descent Model for Burden Distribution in Blast Furnace. J. Iron Steel Res. Int..

[3] Yang Y., Yin Y., Wunsch D., Zhang S., Chen X., Li X., Cheng S., Wu M., Liu K.-Z. (2017). Development of Blast Furnace Burden Distribution Process Modeling and Control. ISIJ Int..

[4] Shen Y., Guo B., Chew S., Austin P., Yu A. (2015). Three-dimensional modeling of flow and thermochemical behavior in a blast furnace. Metall. Mater. Trans. B.

[5] Tian J., Tanaka A., Hou Q., Chen X. (2018). Radar Detection-based Modeling in a Blast Furnace: A Prediction Model of Burden Surface Shape after Charging. ISIJ Int..

[6] Wei J., Chen X., Wang Z., Kelly J., Zhou P. (2015). 3-Dimension Burden Surface Imaging System with T-shaped MIMO Radar in the Blast Furnace. ISIJ Int..

[7] Ho C., Wu S., Zhu H., Yu A., Tsai S. (2009). Experimental and numerical investigations of gouge formation related to blast furnace burden distribution. Miner. Eng..

[8] Teng Z.-J., Cheng S.-S., Du P.-Y., Guo X.-B. (2013). Mathematical model of burden distribution for the bell-less top of a blast furnace. Int. J. Miner. Metall. Mater..

[9] Chen X., Wei J., Xu D., Hou Q., Bai Z. (2012). 3-Dimension Imaging System of Burden Surface with 6-radars Array in a Blast Furnace. ISIJ Int..

[10] Shi L., Wen Y.-B., Zhao G.-S., Yu T. (2016). Recognition of blast furnace gas flow center distribution based on infrared image processing. J. Iron Steel Res. Int..

[11] Xu Y., Wu M., Cao W., Ning Z. (2005). Measurement of the temperature profile based on infrared image processing and its application in blast furnace. Control Eng. China.

[12] Zankl D., Schuster S., Feger R., Stelzer A., Scheiblhofer S., Schmid C.M., Ossberger G., Stegfellner L., Lengauer G., Feilmayr C. (2015). BLASTDAR—A Large Radar Sensor Array System for Blast Furnace Burden Surface Imaging. IEEE Sens. J..

[13] Zankl D., Schuster S., Feger R., Stelzer A. (2017). What a Blast!: A Massive MIMO Radar System for Monitoring the Surface in Steel Industry Blast Furnaces. IEEE Microw. Mag..

[14] Wu C., Wilburn B., Matsushita Y., Theobalt C. (2011). High-quality shape from multi-view stereo and shading under general illumination. Proceedings of the Conference on CVPR 2011.

[15] Wu C., Narasimhan S.G., Jaramaz B. (2010). A multi-image shape-from-shading framework for near-lighting perspective endoscopes. Int. J. Comput. Vis..

[16] Tombari F., Salti S., Di Stefano L. A combined texture-shape descriptor for enhanced 3D feature matching. Proceedings of the 18th IEEE International Conference on Image Processing.

[17] Tombari F., Di Stefano L. (2012). Hough Voting for 3D Object Recognition under Occlusion and Clutter. IPSJ Trans. Comput. Vis. Appl..

[18] Favaro P., Soatto S., Burger M., Osher S. (2008). Shape from Defocus via Diffusion. IEEE Trans. Pattern Anal. Mach. Intell..

[19] Xie W., Nie Y., Song Z., Wang C.C.L. (2019). Mesh-Based Computation for Solving Photometric Stereo with Near Point Lighting. IEEE Comput. Graph. Appl..

[20] Chen Z., Jiang Z., Gui W., Yang C. (2016). A Novel Device for Optical Imaging of Blast Furnace Burden Surface: Parallel Low-Light-Loss Backlight High-Temperature Industrial Endoscope. IEEE Sens. J..

